# Screening and Identification of Novel cGAS Homologues Using a Combination of in Vitro and In Vivo Protein Synthesis

**DOI:** 10.3390/ijms21010105

**Published:** 2019-12-22

**Authors:** Jascha Rolf, Regine Siedentop, Stephan Lütz, Katrin Rosenthal

**Affiliations:** Chair for Bioprocess Engineering, Department of Biochemical and Chemical Engineering, TU Dortmund University, D-44227 Dortmund, Germany; jascha.rolf@tu-dortmund.de (J.R.); regine.siedentop@tu-dortmund.de (R.S.); stephan.luetz@tu-dortmund.de (S.L.)

**Keywords:** cGAS, nucleotidyltransferase, cGAMP, STING, enzyme screening, CFPS, TXTL, heterologous proteins

## Abstract

The cyclic GMP-AMP synthase (cGAS) catalyzes the synthesis of the multifunctional second messenger, cGAMP, in metazoans. Although numerous cGAS homologues are predicted in protein databases, the catalytic activity towards cGAMP synthesis has been proven for only four of them. Therefore, we selected five novel and yet uncharacterized cGAS homologues, which cover a broad range in the field of vertebrates. Cell-free protein synthesis (CFPS) was used for a pre-screening to investigate if the cGAS genes originating from higher organisms can be efficiently expressed in a bacterial expression system. As all tested cGAS variants were expressible, enzymes were synthesized in vivo to supply higher amounts for a subsequent in vitro activity assay. The assays were carried out with purified enzymes and revealed vast differences in the activity of the homologues. For the first time, the cGAS homologues from the Przewalski’s horse, naked mole-rat, bald eagle, and zebrafish were proven to catalyze the synthesis of cGAMP. The extension of the list of described cGAS variants enables the acquisition of further knowledge about the structural and molecular mechanism of cGAS, potentially leading to functional improvement of the enzyme.

## 1. Introduction

**Figure 1:**
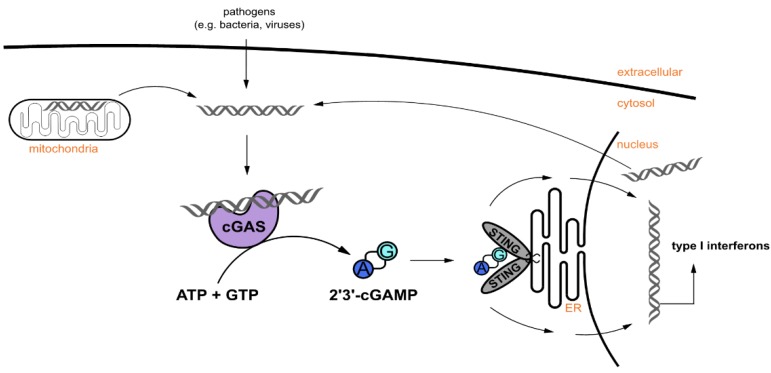
The cyclic GMP-AMP synthase (cGAS) activates the stimulator of interferon genes (STING) pathway. cGAS binds to cytosolic DNA, which can originate from infection with foreign DNA or damaged mitochondria or nuclei. cGAS catalyzes the cyclization of ATP and GTP to cyclic guanosine monophosphate-adenosine monophosphate (2′3′-cGAMP). 2′3′-cGAMP binds to STING that is located at the endoplasmic reticulum (ER) and two cascades are initiated and trigger the transcription of type I interferons.

The cyclic GMP-AMP synthase (cGAS) is a DNA sensor in the cytosol of metazoans [1]. Cytosolic double-stranded DNA (dsDNA) can derive from viruses, bacteria, or the cell itself under stressed conditions and constitutes a danger signal in cells. Upon binding of dsDNA, cGAS undergoes a conformational change that allows access of ATP and GTP into the catalytic pocket. Subsequently, cGAS catalyzes the synthesis of the cyclic dinucleotide (CDN), cyclic guanosine monophosphate-adenosine monophosphate (cGAMP), which acts as a second messenger and activates the stimulator of interferon genes (STING) pathway (Figure 1) [2]. Apart from its role in responding to microbial or viral infections, cGAMP has demonstrated significant antitumor activity in mice and may therefore be used in the immunotherapy of cancer [3,4]. cGAS also participates in cellular senescence and inflammatory diseases [5].

Although several approaches for the chemical synthesis of CDNs and their analogues are established, enzymatic synthesis offers advantageous opportunities. It was shown that cGAMP, produced by cGAS, contains a 2′, 5′ linkage at the GpA position and a 3′, 5′ linkage at the ApG position [6]. This linkage is unique in nature and causes a much greater affinity of 2′3′-cGAMP to STING than the affinity of isomers containing other combinations of phosphodiester linkages [7]. The chemical synthesis of 2′3′-cGAMP contains eight reaction steps and suffers from low yields [8,9], whereas the enzymatic reaction reaches nearly full conversion within a few hours [6]. In recent studies, human cGAS was investigated predominantly with regards to the identification of key residues [10,11] and the kinetic mechanism [12]. Although several homologous enzymes are known, only murine cGAS [6,7], porcine cGAS [10,13], and chicken cGAS [14] received more attention. For the murine and porcine homologue, crystal structures are also available and, in parts, crucial amino acids with relevance for the catalytic function are known. Alignments of amino acid sequences of identified cGAS homologues revealed a low-sequence homology in regions without determined function [10,15]. This high level of variation and the markedly reduced activity of human cGAS in comparison to murine cGAS [11] demonstrate the possibility that other cGAS homologues might be available with enhanced properties or different characteristics and functions. For characterization, cGAS enzymes were synthesized with eukaryotic cell lines or expressed recombinantly in *Escherichia coli.* Eukaryotic genes tend to be difficult to express in bacterial systems, though fusion-tags, such as maltose-binding protein (MBP, 42.5 kDa) [16], glutathione S-transferase (GST, 26 kDa) [2], and small ubiquitin-like modifier (SUMO, 12 kDa) [1], were used for better solubility and functional expression of cGAS. Challenges remain to find suitable expression systems for eukaryotic protein production with good yield and purity, and under conditions conducive to functional protein studies. To realize higher throughput in heterologous protein synthesis, other methods than traditional recombinant expression can be considered.

Cell-free or in vitro protein synthesis (CFPS) is a widely used tool for the rapid transcription and translation of genes with a broad range of applications [17]. It is an attractive alternative to heterologous expression in cell-based systems, which are well established and robust, but laborious and time consuming. CFPS uses the biological machinery without the necessity of living cells and can produce proteins with concentrations at a milligram per milliliter scale from DNA templates within a few hours [18]. The time-consuming steps, such as DNA transformation, culture growth, and induction, can be avoided. Moreover, CFPS is a suitable platform for synthesizing difficult to express proteins, like membrane-anchored proteins [19], proteins with incorporated non-natural amino acids [20], and proteins with a toxic effect on the metabolism of the host cell [21]. Although several CFPS systems are described and have their own advantages, the *E. coli*-based system is the most commonly used due to high protein synthesis rates with high protein yields, streamlined and cost-effective cultivation and extract preparation, and the ability to fold complex proteins. Additionally, this system has been well studied and optimized during recent years [22,23]. *E. coli* is also one of the most used expression strains for cell-based protein synthesis. Although the synthesis of complex proteins derived from higher organisms might be challenging, the applicability of CFPS was already demonstrated in different studies. Mammal proteins were successfully produced with *E. coli*-based CFPS systems, such as therapeutic proteins [24] and human phosphoproteins [25]. The combination of rapid synthesis and the ability to synthesize complex proteins makes CFPS a powerful tool for enzyme screening [26]. Since the synthesis of mammalian proteins is generally possible, CFPS could also be used for the synthesis of cGAS, thereby accelerating the screening of previously unstudied cGAS homologues and elucidating their function.

In this study, different cGAS homologues, which have never been characterized biochemically before, were synthesized with two *E. coli*-based systems and investigated with regard to their ability to produce cGAMP and their corresponding activity in in vitro assays. An *E. coli*-based CFPS system was used to quickly validate the applicability of *E. coli* as an expression host for enzymes originating from various metazoans. We demonstrate that the putative cGAS homologues can be expressed recombinantly in *E. coli* and four previously unknown cGAS variants could be proven to be functional producers of cGAMP. These results extend the list of described cGAS homologues and might contribute to determine the key residues that are responsible for altered functions and can guide protein engineering to evolve cGAS.

## 2. Results and Discussion

### 2.1. Identification of Putative cGAS Homologues in Several Metazoans

The structure of cGAS plays a pivotal role in its function and regulation. Human cGAS (hscGAS) consists of 522 amino acids and can be divided into three parts: The unstructured and positively charged N-terminus, the N-terminal lobe that resembles the nucleotidyltransferase (NTase) fold and partially overlaps with a male abnormal 21 (Mab21) domain, and the C-terminal lobe with a tight five-helix bundle. The unstructured N-terminus does not play an essential role in DNA binding and is dispensable for the catalytic function [1]. Therefore, we decided to focus on truncated sequences, consisting of the NTase and Mab21 domains.

**Figure 2:**
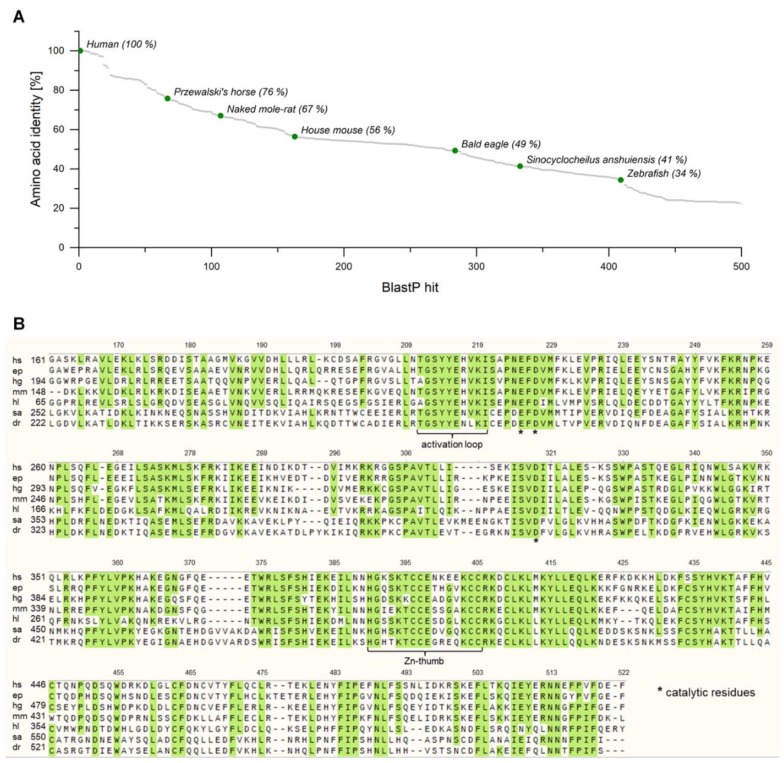
(**A**) BlastP analysis of truncated human cyclic GMP-AMP synthase (cGAS). (**B**) Multiple sequence alignment of truncated amino acid sequences of human (hs), Przewalski’s horse (ep), naked mole-rat (hg), house mouse (mm), bald eagle (hl), *Sinocyclocheilus anshuiensis* (sa), and zebrafish (dr) cGAS. For epcGAS, no information about the full-length sequence is available. Green shading indicates a consensus sequence above a 75% threshold. Numbering above the sequences corresponds to that for hscGAS. The alignment was generated using MUSCLE (SnapGene^®®^, GSL Biotech LLC, Chicago, USA, Version 4.3.10).

In order to identify putative homologues of human cGAS, a BlastP analysis (Basic Local Alignment Search Tool, NCBI, USA) was performed. More than 400 protein sequences with an amino acid identity of more than 30% were retrieved, originating from diverse metazoans (Figure 2A). We selected six proteins with amino acid identities between 34% (homologue from zebrafish) and 76% (homologue from Przewalski’s horse) for further investigation, which covered a broad range in the field of vertebrates (Table A1). The selected sequences coding for cGAS from *Equus przewalskii* (epcGAS), *Heterocephalus glaber* (hgcGAS), *Haliaeetus leucocephalus* (hlcGAS), *Sinocyclocheilus anshuiensis* (sacGAS), and *Danio rerio* (drcGAS) are annotated as predicted cGAS. Additionally, cGAS originating from *Mus musculus* (mmcGAS) was chosen, which was already confirmed to code for a functional NTase that catalyzes the cyclization of ATP and GTP into 2′3′-cGAMP [1]. A multiple sequence alignment for the corresponding, truncated amino acid sequences illustrates the highly conserved regions throughout the homologues (Figure 2B). The amino acid sequences can be found in Supplementary Materials 1. Similar alignments for further cGAS homologues emphasize the conservation in these regions likewise [10,15]. In the following, the numbering of amino acids corresponds to that for hscGAS. The activation loop and the zinc ribbon, two essential motifs for the activity of cGAS, can be found in all sequences as well as the three catalytic residues, which form the active site of the enzyme. Nevertheless, minor differences in these regions of the homologues were apparent so that the enzymes might have different properties, such as activity, stability, or substrate scope.

### 2.2. Expression of cGAS Genes with an E. coli-Based Cell-Free Protein Synthesis System

**Figure 3:**
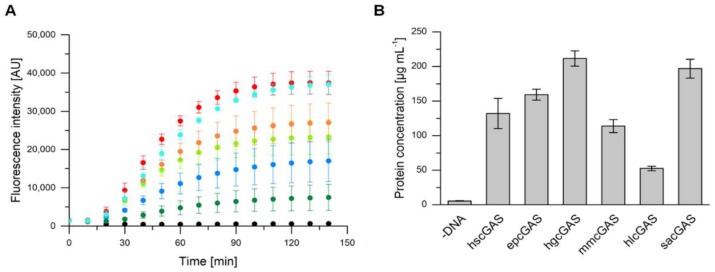
(**A**) Cell-free protein synthesis of superfolder green fluorescent protein (sfGFP)-tagged cyclic GMP-AMP synthases (cGAS) originating from different metazoans performed in a micro plate reader at 30 °C. Black dots represent the negative control, which consisted of protein synthesis mix without a DNA template, light green human (hs), orange Przewalski’s horse (ep), red naked mole-rat (hg), dark blue house mouse (mm), dark green bald eagle (hl), and light blue *Sinocyclocheilus anshuiensis* (sa) cGAS. Error bars relate to biological triplicates. (**B**) Protein concentrations obtained withcell-free protein synthesis (CFPS). Concentrations were calculated based on fluorescence intensity and a standard curve with purified sfGFP. Error bars relate to technical triplicates.

Cell-free protein synthesis can be performed within a few hours and can easily predict the ability of an organism to express certain genes [17]. To investigate if the truncated and non-codon-optimized cGAS genes can be expressed in *E. coli*, we used a self-made *E. coli*-based cell-free protein synthesis system. For convenient online detection of protein synthesis in a microplate reader and to determine concentrations of produced proteins, the variants were fluorescence-tagged. A superfolder variant of green fluorescent protein (sfGFP) was used, whose fusion proteins show proportional fluorescence signal to total protein expression [27]. For all six tested variants an increasing fluorescence signal was detected over a period of about two hours, whereas a negative control without a DNA template did not show any fluorescence throughout the reaction time (Figure 3A). The corresponding protein concentrations were calculated based on the fluorescence measurements and a standard curve with purified sfGFP (Figure 3B). With the exception of hlcGAS, which was obtained with about 50 µg mL^−1^, all other proteins were synthesized with final concentrations between 110 and 210 µg mL^−1^. Although higher protein concentrations were reported for smaller proteins, the achieved concentrations for cGAS variants (about 84 kDa) are reasonable. The highest protein concentration of about 2.3 mg mL^−1^ was published for the synthesis of green fluorescent protein (GFP, 25.4 kDa) with an *E. coli*-based CFPS system [18]. Usually, the amount of available substrate and the accumulation of inhibitory byproducts are the reasons for limited protein titers. In order to avoid these limitations, continuous systems have been established, which, however, suffer from a more complex set-up [28].

The differences in the synthesis rates and in the total protein yield of the six studied homologues can be partially explained with the variability of CFPS by itself [29]. Furthermore, small variations in the template DNA quality can significantly affect the overall synthesis performance [30]. For all homologues, the mean difference in codon usage between their originating species and *E. coli* is in a similar range between 20% and 30% and therefore should have minor effects on variations in protein yield. The bias, however, can decrease the protein synthesis rate. As all tested cGAS variants are expressible in the *E. coli*-based system and an activity assay based on ATP/GTP consumption or cGAMP production is not possible in the complex CFPS mixture, an in vivo expression of the non-sfGFP-labelled proteins and subsequent purification was also attempted for functional studies.

### 2.3. Heterologous Expression in Escherichia coli

**Figure A1:**
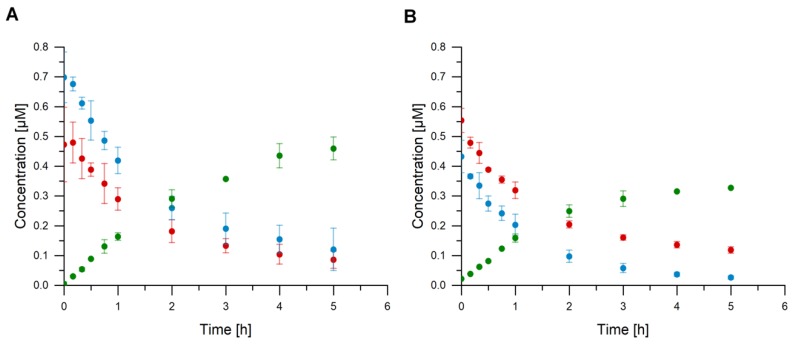
Reaction catalyzed by cGAS shown by GTP (blue), ATP (red), and cGAMP (green) concentrations. **(A)** truncated hscGAS **(B)** truncated hscGAS with sfGFP-tag. Error bars relate to biological triplicates (**A**) or duplicates (**B**).

To synthesize higher amounts for subsequent purification and activity screening, the enzymes were produced in vivo with *E. coli* as the expression strain. The need for the sfGFP-tag was not given in these studies, thus genes without the sfGFP-tag were used for cGAS synthesis. The comparison of the enzyme’s activity with and without the sfGFP-tag revealed no deviation (Figure A1). The gene library was extended with the non-truncated full-length variant of human cGAS (fl hscGAS) and a mutant of mmcGAS. The full-length human cGAS was included because it was expected to have a higher catalytic activity compared to truncated human cGAS [15]. The point mutation of mmcGAS causes the exchange of a highly conserved amino acid located in the Zn-binding motif of the enzyme, probably having a major influence on the catalytic activity.

**Figure 4:**
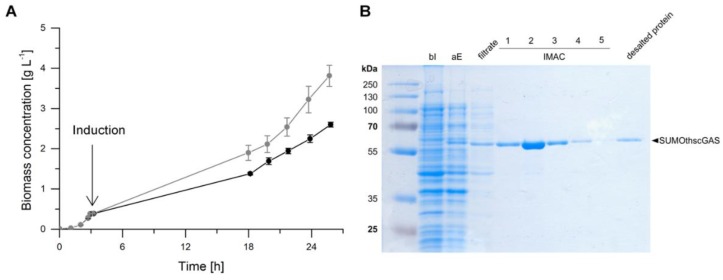
(**A**) Cultivations of induced (black dots) and non-induced (grey dots) *E. coli* BL21 (DE3) pLysS pET-28 a (+) SUMOthscGAS in 2xYT medium and shake flasks. Cells were incubated at 37 °C until an OD_600_ of 1, cooled on ice for 15 min before induction, and subsequently incubated at 15 °C for protein synthesis. A conversion factor of 0.312 g L^−1^ represented by 1 OD_600_ unit was used for calculation of biomass concentration. Error bars relate to biological duplicates. (**B**) SDS-PAGE gel image of synthesis and purification of SUMOthscGAS; bI: before induction; aE: after expression; filtrate: filtered cell lysate; IMAC 1–5: fractions 1–5 of immobilized metal affinity chromatography; desalted protein: eluate after size-exclusion chromatography; Marker: PageRuler^TM^ Prestained Protein Ladder.

Cell growth was monitored exemplarily of *E. coli* BL21 (DE3) pLysS pET-28 a (+) SUMOthscGAS in an induced and non-induced state (Figure 4A). A short lag phase of one hour with negligible cell growth was observed until the exponential growth phase started. At an OD_600_ of 1, the cultures were incubated on ice and subsequently 0.5 mM IPTG were supplemented for induction of heterologous gene expression. Protein synthesis was performed at 15 °C. The induced cultures grew with a slightly lower growth rate of 0.09 ± 0.00 h^−1^ (corresponds to a doubling time of 8.12 ± 0.18 h) than the non-induced cultures (0.10 ± 0.00 h^−1^, doubling time 6.85 ± 0.25 h) and reached lower biomass concentrations within the fermentation time. The expression of heterologous genes is an additional metabolic burden for the production organism and elucidates the observed deterioration in growth [31]. Nevertheless, the growth rates were very similar and a significant negative impact of synthesized cGAS, that could interact with the host cell DNA or compete for substrates with the cell’s metabolism, was not observed. For following experiments, the synthesis was optimized to 20 °C for 11 h and growth rates of 0.17 h^−1^ were obtained. All *E. coli* strains, expressing different cGAS genes, showed sufficient growth in the induced state.

The SDS-PAGE of the synthesis and purification of hscGAS is shown as an example in Figure 4B. The SUMO-tagged variant has a molecular weight of 56 kDa and migrates between 58 and 63 kDa. No significant band is detectable before the induction of protein synthesis. After expression, a band at 60 kDa is visible, which was isolated via the purification procedure. Protein bands were detectable for the other homologues as well (Supplementary Materials 2). Hence, protein synthesis of all nine chosen cGAS variants was considered as successful. To the best of our knowledge, this is the first time that the five predicted cGAS sequences (Przewalski’s horse, naked mole-rat, bald eagle, zebrafish, and *Sinocyclocheilus anshuiensis*) were synthesized. The enzymes were purified, and the obtained concentrations are in a suitable range to analyze their biocatalytic function (Table A2).

### 2.4. cGAS Variants Catalyze cGAMP Synthesis

**Figure 5:**
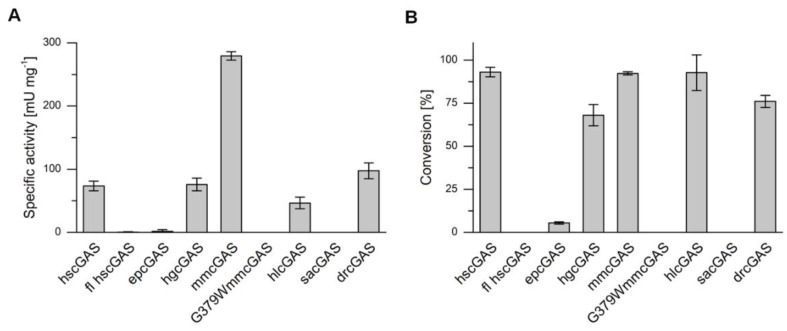
(**A**) Specific activities of cyclic GMP-AMP synthases (cGAS) originating from different metazoans. (**B**) Conversion of ATP by cGAS variants within 24 h. Human (hs), full-length human (fl hs), Przewalski’s horse (ep), naked mole-rat (hg), house mouse (mm), house mouse with amino acid exchange (mmG379W), bald eagle (hl), *Sinocyclocheilus anshuiensis* (sa), and zebrafish (dr) cGAS. In vitro activity assays were performed at 37 °C with 40 μg mL^−1^ cGAS, 0.1 mg mL^−1^ HT-DNA, and 0.5 mM ATP and GTP, respectively. Error bars relate to biological triplicates.

Specific activities of cGAS variants were determined by in vitro assays and RP-HPLC measurements of cGAMP production (Figure 5, Supplementary Materials 2). The cGAS variants had not only diverse specific activities, but also differences in the maximal substrate conversion within 24 h (Table A3). While some homologues did not catalyze cGAMP synthesis, others showed nearly full substrate conversion, such as hscGAS, mmcGAS, and hlcGAS. For all active cGAS variants, HPLC measurements confirmed the formation of cGAMP and the absence of the homo-dinucleotides c-di-GMP and c-di-AMP. Other NTases, such as DncV, which is a bacterial cGAS homologue originating from *Vibrio cholerae*, produce these homo-dinucleotides as typical by-products [32]. Although cGAS was studied extensively in the last years, only a few enzyme parameters for cGAS were described. The achieved turnover numbers (k_cat_) for homologues were between 0.001 ± 0.001 s^−1^ (for fl hscGAS) and 0.52 ± 0.01 s^−1^ (for mmcGAS) (Table A3), and are at a comparable scale with published k_cat_ values, which range from 0.003 [6] to 0.06 s^−1^ [33] for the human homologue. The differences are explainable with variations in the setup of the activity assay, such as higher substrate concentrations.

The zebrafish homologue has the lowest amino acid identity to hscGAS of less than 35%. Nevertheless, similar activities were determined. In contrast, the enzyme of the significantly closer related cave fish *Sinocyclocheilus anshuiensis* lacked any catalytic activity under the tested conditions. It can be assumed that the temperature optima of hlcGAS, drcGAS, and sacGAS are distinct to the assay temperature of 37 °C, because the body temperatures of the originating organisms are considerably lower. Lowering the assay temperature could therefore increase the activity of the enzymes. Moreover, full-length hscGAS was significantly less active than the truncated version. This finding is contrary to previous studies, which stated an enhancement of enzyme activity due to the highly charged N-terminus of cGAS [15,34]. This might indicate incorrect folding of the full-length enzyme during heterologous gene expression and therefore lowered the total concentration of active enzyme in the activity assay. In the case of epcGAS, the missing N-terminus might be an explanation for the low activity and conversion. Despite the taxonomic vicinity of epcGAS to hscGAS, it produced only marginal cGAMP concentrations. The full-length epcGAS variant has not been published in protein databases up to now; though it cannot be excluded that enhancing motifs are located in the N-terminus.

The cGAS homologue of *Sinocyclocheilus anshuiensis* and the mutant of *Mus musculus* (G379W) did not convert any ATP and GTP to cGAMP, whereas the full length human cGAS and the enzyme of the Przewalski’s horse showed marginal activities and conversion of the substrates. The mutation of mmcGAS is positioned in the zinc thumb and resulted in the loss of enzyme activity, which can be explained by disruption of proper folding of the enzyme and ablation of the zinc-binding capacity. Substitutions of other amino acids in the zinc-coordination site near the DNA-binding cleft already confirmed the need of this motif for the enzyme’s function [10,16]. The wild-type mmcGAS showed the highest specific activity of 279.2 ± 6.7 mU mg^−1^ with almost full conversion. The determined catalytic activity of hscGAS and mmcGAS is slightly lower compared to already published data, which might be attributed to differing activity assay compositions with regard to buffer and used DNA for cGAS activation [11]. Nevertheless, we observed a four-fold higher activity of mmGAS in comparison to the human enzyme, supporting previous findings. Zhou et al. performed an enzyme kinetics analysis of both enzymes and achieved significantly higher activities for mmcGAS [11]. The responsible amino acids causing this difference were identified; N187 and R195 of mmcGAS stabilizes the interaction of the enzyme and DNA by improving the contact to the DNA phosphate backbone and increasing the overall positive charge of the DNA-binding surface. The exchange of both amino acids (K187N/L195R) in the catalytic domain of hscGAS increased the activity to similar levels compared to mmcGAS. It has also been shown that the replacement of a single amino acid does not affect the enzyme’s activity. In our study, the asparagine (N) at position 187 is highly conserved for all homologues except for hscGAS with a lysine (K) at position 187 (Figure 2B). Interestingly, position 195 is not conserved between the homologues and two other amino acids, alanine (A) and glutamine (Q), a hydrophobic and a polar amino acid, respectively, were found. The homologues epcGAS, hgcGAS, and hlcGAS, have a common glutamine at position 195, though showing different activities. Obviously, other amino acids also play a major role for the enzyme’s activity. A recent study highlighted another cGAS-DNA interface, which is formed by an extended basic patch of positively charged residues originating from the α-region (Q264, K275, K279, K282, and K285), the KRKR-motif (K299, R300, K301, and R302), and the KKH-loop (K427, K428, and K432) [35]. Whereas the residues in the α-region and in the KRKR-motif are mainly conserved throughout the investigated homologues, the KKH-loop is completely inhomogeneous and could explain varying enzyme activities.

To sum up, in previous studies, human, murine, and porcine cGAS were already expressed heterologously in *E. coli* strains and activity assays were conducted with purified enzymes. In these assays, significantly different activities were described for the three homologues. We extended the range of recombinant synthesized cGAS variants by five homologues. Four of them, epcGAS, hlcGAS, hgcGAS, and drcGAS, could be proven to be functional cGAS catalyzing the synthesis of cGAMP for the first time. As already described, the specific activity varies significantly between the homologues and a rational determination of key residues is still challenging. Although the homologue with the highest activity, mmcGAS, was already known, these results demonstrate the potential of screening of cGAS homologues for biotechnological application.

## 3. Materials and Methods

### 3.1. Strains and Plasmids

*E. coli* BL21 (DE3) and *E. coli* BL21 (DE3) pLysS were used for heterologous expression. *E. coli* DH5α was used for molecular cloning. The amino acid sequences of cGAS homologues and their corresponding nucleotide sequences were obtained from online databases (Table A1). The template pET-28 a (+) SUMOflhscGAS was cloned using the restriction sites *Nde*I and *Hind*III. From this template, the truncated human cGAS (thscGAS) and the SUMO sequence were amplified and fused with appropriate primers. The insert and vector pET-28 a (+) were cloned following the restriction ligation protocol with *Hind*III and *Nde*I as restriction sites. The gene sequences were synthesized by Eurofins and other homologues than hscGAS were cloned into pET-28 a (+) SUMOthscGAS using *Afl*II and *Xho*I restriction sites or Gibson Assembly [36]. For construction of fusion proteins with superfolder green fluorescent protein (sfGFP), truncated SUMOcGAS genes were amplified with appropriate primers and cloned into pET GFP LICs cloning vector (linearized with *EcoR*V-HF) via Gibson Assembly or sequence and ligation-independent cloning (SLIC) [37]. The vector pET GFP LIC (u-msfGFP) was a gift from Scott Gradia (RRID:Addgene_29772; http://n2t.net/addgene:29772). The resulting plasmids were prepared using the NucleoSpin^®^ Plasmid (NoLid) Kit (Macherey-Nagel, Düren, Germany) for transformation of *E. coli.* For CFPS, plasmids were prepared using the GeneJET Plasmid Midiprep Kit (ThermoFisher Scientific, Waltham, MA, USA), followed by a second purification with the NucleoSpin^®^ Gel and PCR Clean-up Kit (Macherey-Nagel, Düren, Germany). A list of plasmids used in this study is shown in Table 1 and nucleotide sequences of the genes and primers are provided in Supplementary Materials 1.

### 3.2. E. coli Extract Preparation

The *E. coli* extract was prepared as described by Levine et al. [30] with some modifications, which are stated in the following. *E. coli* BL21 (DE3) was transformed with pAR1219 [38] for overexpression of T7 RNA polymerase (T7RNAP). A preculture of 50 mL LB (10 g L^−1^ tryptone, 5 g L^−1^ yeast extract, 5 g L^−1^ NaCl) medium with 100 µg mL^−1^ ampicillin was inoculated with a single colony of *E. coli* BL21 (DE3) pAR1219. The preculture was grown for 16 h at 200 rpm and 37 °C. The main culture of 500 mL 2xYTPG (16 g L^−1^ tryptone, 10 g L^−1^ yeast extract, 5 g L^−1^ NaCl, 7 g L^−1^ K_2_HPO_4_, 3 g L^−1^ KH_2_PO_4,_ 18 g L^−1^ glucose) medium in a 2-L baffled shake flask was inoculated to an OD_600_ of 0.1 and grown at 200 rpm at 37 °C. At an OD_600_ of 0.6, 1 mM of isopropyl-β-D-thiogalactopyranoside (IPTG, Carl Roth, Karlsruhe, Germany) was added to induce T7RNAP production. Cells were harvested at an OD_600_ of 3 and pelleted via centrifugation at 5000 *g* for 10 min at 10 °C. The pellets were washed three times with 4 °C cold S30 buffer (10 mM tris acetate, pH 8.2; 14 mM magnesium acetate; 60 mM potassium acetate; and 2 mM dithiothreitol (DTT, Carl Roth, Karlsruhe, Germany), flash-frozen with liquid nitrogen, and stored at −80 °C. For lysis, cells were thawed on ice and resuspended in 1 mL of S30 buffer per gram wet cells. Three cycles of sonication were performed for 40 s and 2 mM DTT were added. Cellular debris was removed by centrifugation at 18,000 g for 10 min at 4 °C. The supernatant was incubated at 450 rpm for 60 min at 37 °C, and then centrifuged at 10,000 g for 10 min at 4 °C. The final supernatant was flash-frozen with liquid nitrogen and stored at –80 °C until use. Protein concentrations were determined by the Bradford method using bovine serum albumin (BSA, manufacturer, city, state abbreviation if USA or Canada, country) as a standard [39]. Obtained extracts contained between 40 and 60 mg mL^−1^ total protein.

### 3.3. Cell-Free Protein Synthesis (CFPS)

CFPS reactions with a reaction volume of 10 µL were performed in a 384-well microplate containing: 9.6 to 14.4 mg mL^−1^ protein (from extract), 10 mM magnesium glutamate, 130 mM potassium glutamate, 1.5 mM each of 20 amino acids (except leucine), 1.25 mM leucine, 50 mM 4-(2-hydroxyethyl)-1-piperazineethanesulfonic acid (HEPES), 1.5 mM ATP and GTP, 0.9 mM cytidine triphosphate (CTP) and uridine triphosphate (UTP), 0.2 mg mL^−1^ *E. coli* tRNA, 0.26 mM coenzyme A (CoA), 0.33 mM nicotinamide adenine dinucleotide (NAD), 0.75 mM cyclic adenosine monophosphate (cAMP), 0.068 mM folinic acid, 1 mM spermidine, 30 mM 3-phosphoglyceric acid (3-PGA), 2 % polyethylene glycol-8000 (PEG), and 1 nM plasmid DNA. Reactions were incubated for 6 h at 30 °C in a FLUOstar^®®^ Omega multi-mode microplate reader (BMG LABTECH, Ortenberg, Germany) and fluorescence signals were measured with λ_Ex_ 485 nm and λ_Em_ 520 nm.

### 3.4. Cell-Based Protein Synthesis

A preculture of 5 mL 2xYT (16 g L^−1^ tryptone, 10 g L^−1^ yeast extract, 5 g L^−1^ NaCl) medium with 50 µg mL^1^ kanamycin and 25 µg mL^−1^ chloramphenicol was inoculated with a single colony of *E. coli* BL21 (DE3) pLysS pET-28 a (+) SUMOtcGAS. The preculture was grown for 9 h at 200 rpm and 37 °C. The main culture of 100 mL 2xYT medium was inoculated in a 1-L baffled shake flask to an OD_600_ of 0.05 and grown at 200 rpm at 37 °C. Once an OD_600_ of 1 was reached, heterologous gene expression was induced with 0.5 mM IPTG. The culture was incubated at 20 °C and 200 rpm for 11 h. Samples for SDS-PAGE were taken before induction and before harvesting. Cells were harvested in 25-mL aliquots by centrifugation at 4600 *g* for 40 min at 4 °C and the pellets were stored at –20 °C.

For cell disruption, the pellets were thawed on ice and resuspended in 10 mL lysis buffer (50 mM Tris-HCl (pH 8), 300 mM NaCl, 40 mM imidazole, 1 mM TCEP). Five cycles of sonication were performed for 30 s. Cellular debris was removed by two rounds of centrifugation at 43,000 x g for 15 min at 4 °C. Soluble components were sterile filtered (0.2 μm) before further procedures. The supernatant obtained was used to purify cGAS variants using nickel affinity columns (HisTrap FF crude, GE Healthcare, Solingen, Germany). The lysis buffer was used as loading buffer and cGAS was eluted with 6 mL of elution buffer (20 mM Tris-HCl (pH 7.4), 150 mM NaCl, and 300 mM imidazole). Protein concentrations were determined and the three fractions with the highest concentrations were pooled to 2.5 mL of volume. Buffer exchange was performed with activity buffer (40 mM HEPES (pH 7.2), 10 mM MgCl_2_) by size-exclusion chromatography using a PD-10 SephadexTM G-25 column (GE Healthcare, Little Chalfont, UK). The purity of the protein was judged by 12% sodium dodecyl sulfate polyacrylamide gel-electrophoresis (SDS-PAGE).

### 3.5. Activity Measurement

Enzyme activity assays were performed with purified and desalted protein in 2-mL micro tubes and 2-mL reaction volume. The assays were carried out in activity buffer with 40 μg mL^−1^ His_6_SUMOtcGAS, 0.1 mg mL^−1^ herring testis DNA (HT-DNA), and 0.5 mM ATP and GTP, respectively. The assays were incubated at 300 rpm and 37 °C. Samples were taken after 0, 10, 20, 30, and 45 min as well as 1, 2, 3, 4, 5, and 24 h. Samples were heat inactivated at 95 °C for 5 min.

### 3.6. Quantification of cGAMP, ATP, and GTP

Reverse-phase high performance liquid chromatography (RP-HPLC) was performed to elucidate the enzyme activity by quantification of ATP, GTP, and cGAMP. The method was developed on the basis of Liu et al. [40]. A Knauer Azura HPLC system (KNAUER GmbH, Berlin, Germany) consisting of an autosampler (AS 6.1L), pump system (P 6.1L), column oven (CT 2.1), and a wavelength detector (MWD 2.1L) was connected to an ISAsphere 100-5 C18 AQ column (250 × 4 mm, Isera, Düren, Germany), heated to 30 °C. Then, 5-μL sample were injected and eluted with a gradient of mobile phase A (0.06 mol L^−1^ K_2_HPO_4_ and 0.04 mol L^−1^ KH_2_PO_4_, pH 7.0, adjusted with KOH) and mobile phase B (95% acetonitrile) with a flow rate of 1 mL min^−1^ (0 min 100% A, 0% B; 3 min 100% A, 0% B; 5 min 95% A, 5% B; 8 min 75% A, 25% B; 14 min 60% A, 40% B; 17 min 100% A, 0% B; 20 min 100% A, 0% B).

### 3.7. Verification of cGAMP Production

Liquid chromatography-mass spectrometry (LC-MS) with an electrospray ionization (ESI) interface was used to verify the production of cGAMP. The Agilent Technologies 1260 Infinity II LC System was coupled to Agilent Technologies 6120 Single Quadrupole LC/MS System (Agilent Technologies Inc., Santa Clara, CA, USA) and further equipped with a Diode Array Detector (1260 DAD HS, Agilent Technologies Inc., Santa Clara, CA, USA), Multicolumn Thermostat (1260 MCT, Agilent Technologies Inc., Santa Clara, CA, USA), Degasser (1260 Degasser, Agilent Technologies Inc., Santa Clara, CA, USA), Multisampler (1260 Multisampler), Binary Pump (1260 Binary Pump, Agilent Technologies Inc., Santa Clara, CA, USA) and an Agilent Poroshell 120 EC-C18 (4.6 × 100 mm, 2.7 μm particle size) column. Sample volumes of 5 μL were injected and separated by a gradient of mobile phase A (0.1 % formic acid) and mobile phase B (acetonitrile) (0 min 5% A, 95% B; 7 min 95% A, 5% B; 9 min 5% A, 95% B; 14 min 5% A, 95% B). The flow rate was set to 1 mL min^−1^ at a column temperature of 40 °C and a detection wavelength of 254 nm. Measured masses ranged from 100 to 1000 m/z. The gas chamber was heated to 350 °C with a gas flow of 12 L min^−1^. The capillary voltage was set to 3 kV and the nebulizer pressure to 35 psi.

## 4. Conclusions

*E. coli* is the most popular expression organism for the synthesis of recombinant proteins. It has become a well-established cell factory with many molecular tools and protocols for pro- and eukaryotic protein production. However, proteins with molecular weights above 60 kDa tend to be difficult to express [41]. Furthermore, codon bias, protein folding, and post-translational modifications are the major obstacles preventing prokaryotic expression systems being used for eukaryotic protein synthesis. The synthesis of cGAS might therefore demonstrate a challenge, as these are eukaryotic enzymes originating from metazoans and are moreover regulated by post-translational modifications [42,43]. In this study, we used a CFPS system to elucidate the applicability of *E. coli* as an expression host for the synthesis of a variety of cGAS homologues. CFPS systems reduce cells to the basic function of protein formation and are versatile tools to investigate gene expression and protein synthesis. All of the investigated homologues were successfully synthesized using this pre-screening approach. In addition, the transfer from in vitro to in vivo expression was accomplished; six novel and three already known enzymes were available in purified form for further investigations. For the first time, the cGAS homologues from the Przewalski’s horse, naked mole-rat, bald eagle, and zebrafish were proven to catalyze the conversion of ATP and GTP into cGAMP. In the future, the gained knowledge will contribute to estimate the relevance of specific amino acid residues important for cGAS functionality. In addition, the understanding of structural and molecular mechanisms of protein function will facilitate the development of tailored enzymes with, e.g., higher activity, stability, or modified reaction scope.

## Figures and Tables

**Table 1:** Plasmids used in this study.

Plasmid	Encoded Protein	Source
pET-28 a (+) SUMOthscGAS	truncated hs cGAS (N-terminal His_6_SUMO)	this study
pET-28 a (+) SUMOflhscGAS	full-length hs cGAS (N-terminal His_6_SUMO)	this study
pET-28 a (+) SUMOtepcGAS	truncated ep cGAS (N-terminal His_6_SUMO)	this study
pET-28 a (+) SUMOthgcGAS	truncated hg cGAS (N-terminal His_6_SUMO)	this study
pET-28 a (+) SUMOtmmcGAS	truncated mm cGAS (N-terminal His_6_SUMO)	this study
pET-28 a (+) SUMOtmm_G379W_cGAS	truncated mm_G379W_ cGAS (N-terminal His_6_SUMO)	this study
pET-28 a (+) SUMOthlcGAS	truncated hl cGAS (N-terminal His_6_SUMO)	this study
pET-28 a (+) SUMOtsacGAS	truncated sa cGAS (N-terminal His_6_SUMO)	this study
pET-28 a (+) SUMOtdrcGAS	truncated dr cGAS (N-terminal His_6_SUMO)	this study
pET SUMOthscGASsfGFP	truncated hs cGAS (N-terminal His_6_SUMO, C-terminal sfGFP)	this study
pET SUMOtepcGASsfGFP	truncated ep cGAS (N-terminal His_6_SUMO, C-terminal sfGFP)	this study
pET SUMOthgcGASsfGFP	truncated hg cGAS (N-terminal His_6_SUMO, C-terminal sfGFP)	this study
pET SUMOtmmcGASsfGFP	truncated mm cGAS (N-terminal His_6_SUMO, C-terminal sfGFP)	this study
pET SUMOthlcGASsfGFP	truncated hl cGAS (N-terminal His_6_SUMO, C-terminal sfGFP)	this study
pET SUMOtsacGASsfGFP	truncated sa cGAS (N-terminal His_6_SUMO, C-terminal sfGFP)	this study
pET GFP LIC (u-msfGFP)	sfGFP	Addgene_29772
pAR1219	T7 RNA polymerase	[38]

## Supplementary Materials

Supplementary materials can be found at https://www.mdpi.com/1422-0067/21/1/105/s1.

## Abbreviations

BlastP	Basic local alignment search tool
CDN	Cyclic dinucleotide
CFPS	Cell-free protein synthesis
cGAMP	Cyclic guanosine monophosphate-adenosine monophosphate
cGAS	cyclic guanosine monophosphate-adenosine monophosphate synthase
dr	*Danio rerio*
ER	Endoplasmic reticulum
ep	*Equus przewalskii*
GFP	Green fluorescent protein
GST	Glutathione S-transferase
hg	*Heterocephalus glaber*
hl	*Haliaeetus leucocephalus*
hs	*Homo sapiens*
IMAC	Immobilized metal affinity chromatography
Mab21	Male abnormal 21
MBP	Maltose binding protein
mm	*Mus musculus*
NTase	Nucleotidyltransferase
sa	*Sinocyclocheilus anshuiensis*
sfGFP	Superfolder green fluorescent protein
STING	Stimulator of interferon genes
SUMO	Small ubiquitin-like modifier

## Appendix A

**Table A1:** References for protein and nucleotide sequences of cGAS homologues.

Metazoan	Binomial Name	Abbreviation	Protein Reference Sequence	Nucleotide Reference Sequence
Human	*Homo Sapiens*	hs	NP_612450.2	NM_138441.3
Przewalski’s horse	*Equus przewalskii*	ep	XP_008535543.1	XM_008537321.1
Naked mole-rat	*Heterocephalus glaber*	hg	XP_010564126.1	XM_021243735.1
House mouse	*Mus musculus*	mm	XP_016348574.1	NM_173386.5
Bald eagle	*Haliaeetus leucocephalus*	hl	XP_021099394.1	XM_010565824.1
-	*Sinocyclocheilus anshuiensis*	sa	NP_775562.2	XM_016493088.1
Zebrafish	*Danio rerio*	dr	XP_016348574.1	XM_680019.5

**Table A2:** Biomass-specific protein yield of heterologous expression of cGAS homologues in *Escherichia coli.*

cGAS Variant	Protein Yield [mg g_CDW_^−1^]
hs	6.8
fl hs	6.7
ep	3.0
hg	9.9
mm	13.6
mm(G379W)	21.5
hl	4.1
sa	9.8
dr	3.2

**Table A3:** Enzyme kinetic parameters of cGAS variants. cGAS enzyme activity was calculated based on cGAMP formation. Conversion is shown for ATP. Data are represented as mean ± SD of biological triplicates.

cGAS variant	Specific Activity [mU mg^−1^]	k_cat_ [s^−1^]	Conversion [%]
hs	73.5 ± 7.8	0.14 ± 0.01	93.0 ± 2.8
fl hs	0.5 ± 0.5	9.3 × 10^−4^ ± 9.3 × 10^−4^	n.d.
ep	1.7 ± 2.6	3.1 × 10^−3^ ± 4.8 × 10^−3^	5.5 ± 0.7
hg	75.8 ± 10.0	0.14 ± 0.02	68.0 ± 6.2
mm	279.2 ± 6.7	0.52 ± 0.01	92.3 ± 0.9
mm(G379W)	n.d.	n.d.	n.d.
hl	46.6 ± 9.2	0.09 ± 0.02	92.7 ± 10.3
sa	n.d.	n.d.	n.d.
dr	97.4 ± 12.6	0.18 ± 0.02	76.0 ± 3.5

n.d.: not detected.
